# A Novel Transcatheter Device for the Edge-to-Edge Treatment of Tricuspid Regurgitation: A Preliminary Evaluation

**DOI:** 10.1007/s10439-023-03399-4

**Published:** 2023-11-07

**Authors:** Eleonora Salurso, Francesca Perico, Fabio Pappalardo, Marco Gard, Matteo Antoniotti, Eugenio Passanante, Daniele Zanotti, Michele De Bonis, Ottavio Alfieri, Riccardo Vismara

**Affiliations:** 1 Department of Electronics, Information and Bioengineering, Politecnico di Milano, Via Golgi 39, 20133 Milan, Italy; 2 Gard Consulting, Ivrea, Turin Italy; 3 StarTric s.r.l., Milan, Italy; 4 Department of Cardiac Surgery, San Raffaele University Hospital, Milan, Italy

**Keywords:** Tricuspid regurgitation, Percutaneous treatments, Clover technique

## Abstract

Tricuspid regurgitation (TR) is the most common pathology of the tricuspid valve (TV), with significant mortality in severe cases. A well-established strategy to treat TR is represented by the clover surgical technique, which consists of stitching together the free edges of TV leaflets, producing a clover-shaped valvular orifice. Transcatheter treatments for TR constitute a valuable alternative for high-risk patients. In this work we investigated haemodynamic performances and safety of a novel device (StarTric device (STD)) aiming to perform the clover technique via percutaneous access. To assess haemodynamic performances, STD and clover were applied on porcine pathological TVs and tested. Fluid dynamic indexes of both strategies were compared to the pathological model. To evaluate device safety, forces exchanged between device and leaflets were compared to the extraction force (EF) required to STD to completely pass through the leaflet. Clover technique and STD induced a comparable TV backflow reduction (48% and 47%, respectively), with associated increase of TV flow in all tested conditions. Diastolic transvalvular pressure similarly increased indicating a reduction, though not significant, of the valvular orifice. Forces ranged from 1N to 1.71N, compared to an EF of 22.16 ± 8.6N. Force varied significantly amongst different working conditions (normotensive, mild, and severe hypertensive) for each leaflet, whilst no significative variation was found on different leaflets in the same working condition. In the adopted experimental scenario, STD demonstrated comparable efficacy to the surgical strategy in restoring TV haemodynamic. The forces acting on the leaflets following STD implantation were far lower when compared to EFs.

## Introduction

Tricuspid regurgitation (TR) is the most common disease of the tricuspid valve (TV), with a mortality rate of 45.6% for severe cases [1]. TR can be characterized as organic or functional (FTR) based on the aetiology, with FTR being the more common cause (90%) [2]. The gold standard of surgical repair for TR is represented by annuloplasty, yet the efficacy of this procedure is reduced in case of progressive dilation of the right ventricle (RV) [3] or in presence of leaflet prolapse or flail occurring in post-traumatic or severe degenerative TR. In these cases, additional surgical strategies are needed together with the annuloplasty.

Amongst these strategies the Clover technique represents a well-established option. This technique consists of stitching together the middle point of the free edges of the tricuspid leaflets, producing a clover-shaped valvular orifice [4]. The efficacy of the technique, in case of complex TV lesions either post-traumatic or degenerative, was reported with low recurrence of severe TR (at 12-year follow-up 77.5% of patients had no or mild TR [5]). The technique was also applied to a small pool of patients with FTR, suggesting the possibility to widen the set of treatable patients.

Despite the association of severe TR with poor survival, relatively few patients undergo TV surgery. Patient at elderly ages or with comorbidities are limited by the high in-hospital mortality rates [6, 7] and are mostly managed pharmacologically only to improve symptoms. A promising alternative for these patients is represented by transcatheter approaches which aim to reduce the invasiveness of the conventional surgery, reducing the in-hospital stay and widen the number or treatable patients.

The advancements in the field of transcatheter technology of the last decades have led to the development of novel percutaneous strategies to treat valvular diseases, including TR. First-generation TV solutions, which are nowadays the most advanced in terms of clinical validation, are adaptations of existing mitral valve solutions to the right side. As an example amongst the edge-to-edge devices, the Pascal device was adopted for the treatment of TV in patient with severe TR [8] or the Triclip system [9], a new iteration of the MitraClip device, has been developed with a delivery system designed for the TV anatomy. Though, the necessity to address the specific morphology of the TV-RV complex is moving the focus to new solutions developed for the TV. In this setting, a novel transcatheter device, the Startric device (STD), is under development with the aim to replicate the clover surgical technique via percutaneous access. The main innovation provided by this technology lies in the ability to grasp the three TV leaflets in a single procedure. This is thought to confer, for specific pathological anatomies, benefits in terms of procedural time and efficacy.

In this work, we investigated a prototype replicating the main functional features of the implantable components underlying the STD technology. Tests were performed on an experimental platform reproducing FTR. We assessed the haemodynamic efficacy of STD in reducing TR and we performed a preliminary evaluation of STD interaction with biological structures.

Specifically, the haemodynamic performance of STD was assessed comparing fluid dynamic behaviour of TV treated with STD and with Clover technique in absence of annuloplasty. The safety of the system was investigated comparing the force exchanged between STD and leaflets to the extraction force, defined as the force required to STD to completely pass through the leaflet.

## Materials and Methods

### Device Prototype

**Fig. 1:**
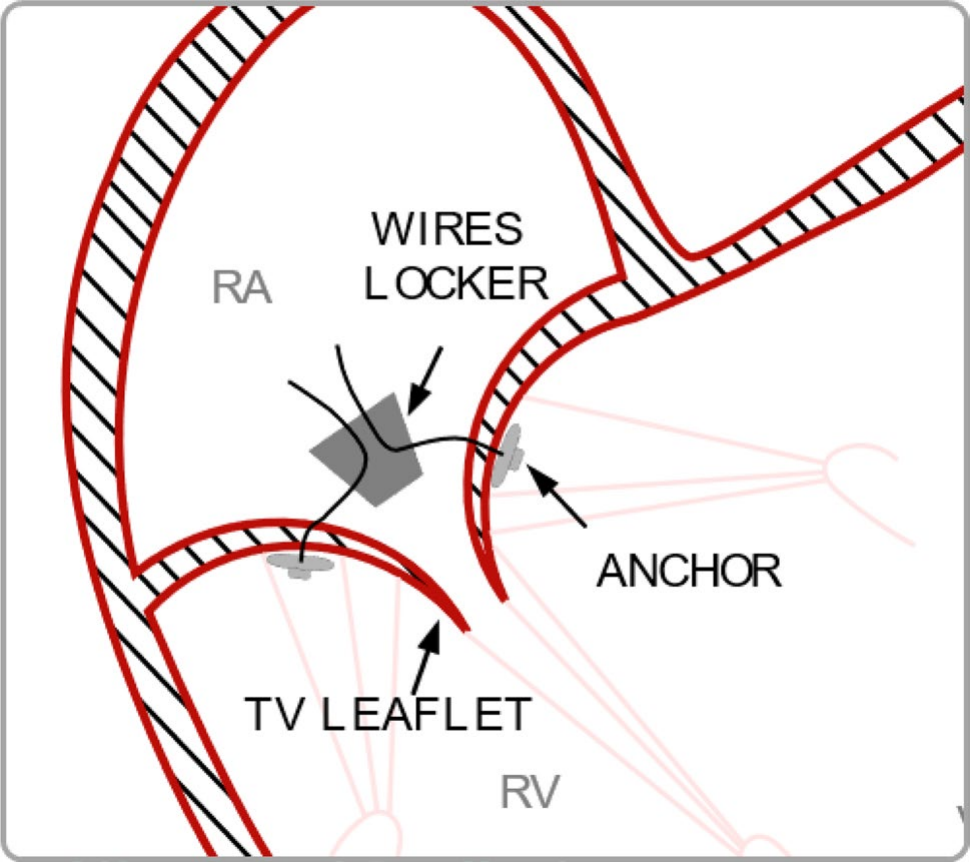
Schematic representation of device implant. *RA* right atrium; *RV* right ventricle, *TV* tricuspid valve

The prototype of the device is composed by three suture wires featuring anchors operating as pledgets on the leaflets ventricular surface and a wire locker aimed at wires’ tension adjustment and blockage. A schematic representation of device implant is reported in Fig. 1. The wire of each anchor was passed through one of the TV leaflets, approximately 5 mm far from the free edge. The wires were then passed through the wire locker, in the atrium.

### Heart Samples Selection and Preparation

A total of 21 porcine heart samples were selected according to the following protocol. Valve samples were sized by means of annuloplasty sizer, and annular dimension was used as a selection criterion. Samples with antero-posterior diameter around 40 mm, when subjected to a 20-mmHg ventricular pressure, were selected for the tests. Selection was made on the basis that a TV diameter > 40 mm is associated to significant TV regurgitation in human patients [10].

Hearts were collected from a local abattoir and stored at − 30 °C for three days before use. Samples were defrosted at room temperature for 24 h and then prepared.

In order to be connected to the pulsatile setup, 15 hearts (six for the haemodynamic assessment and nine for the interaction force tests) were prepared as follows. Left ventricle was dissected and coronary sinus was closed with a suture. Hydraulic connectors were secured at the level of the inferior vena cava (atrial connector), pulmonary artery (pulmonary connector), and through an opening created on the septal wall (septal connector). The right atrium was cut open at the level of the superior vena cava and the opening was secured with a cable tie to a custom 3D-printed *cables connector*, designed for endoscopic camera insertion and for the exit of load cells electric cables and device suture wires. The remaining 6 hears were exploited for the extraction force tests excising the tricuspid valve and isolating the single leaflets.

### Pathological Ex Vivo Model of FTR and Pulsatile Flow Mock Loop

**Fig. 2:**
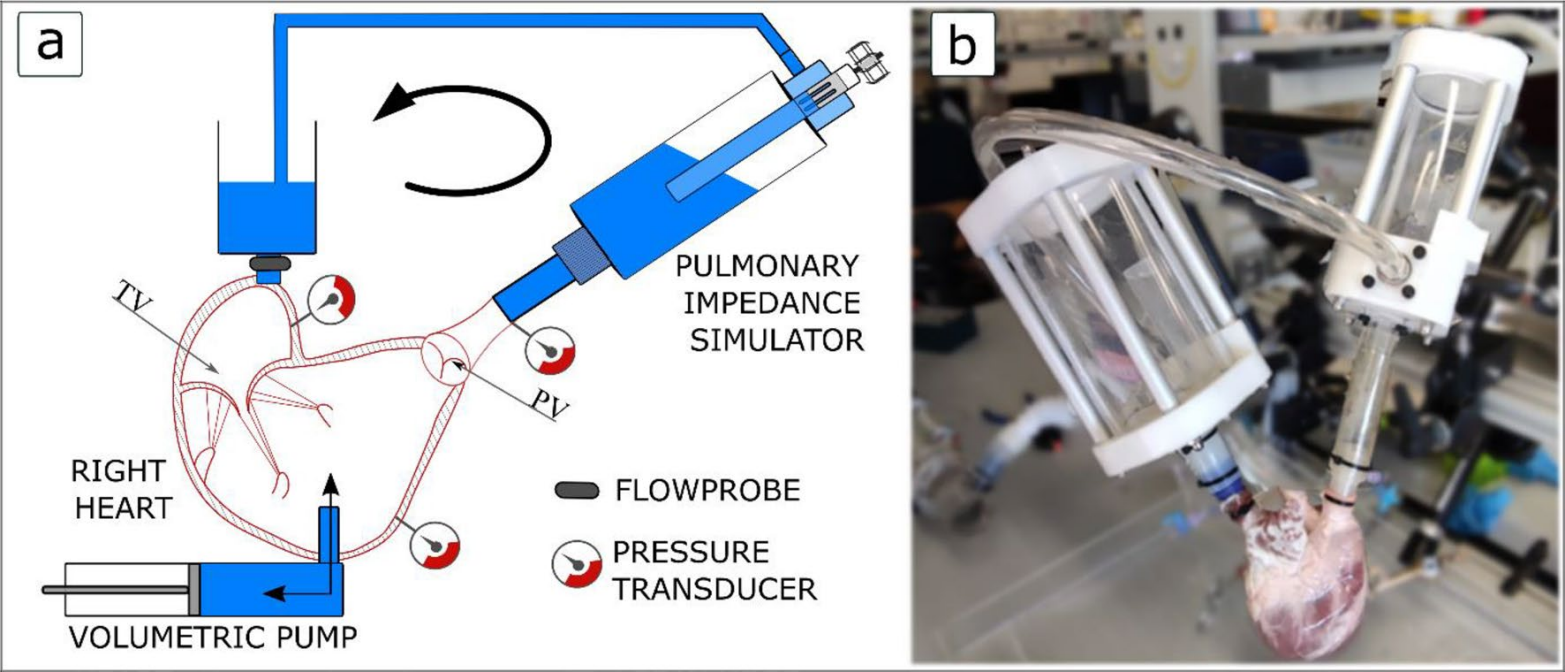
**a** Scheme of the pulsatile flow mock loop. *TV* tricuspid valve, *PV* pulmonary valve; **b** picture of a heart sample connected to afterload and preload

Tests were conducted on an ex vivo model of FTR reproduced in a pulsatile right circulation simulator. The pathological model replicating the mechanistic aspects of FTR was obtained in defrosted porcine heart samples, prepared as above reported, due to spontaneous dilation of right heart once subjected to physiological pressures. The pathological model featured tricuspid valve annulus enlargement, leaflets tethering, right ventricle dilation resulting in TV malcoaptation, and associated tricuspid regurgitation [11–13]. Each heart sample was housed in a pulsatile mock loop which scheme is reported in Fig. 2.

The sample was connected to a volumetric pump via septal connector. The pump [14] reproduced right ventricle waveforms. During systole, the working fluid (distilled water) was pushed from the pump head into the ventricle and, through the pulmonary valve and artery, into a pulmonary impedance simulator. The impedance simulator, based on a RCR Windkessel model, comprised a characteristic resistance, a compliance chamber, and an adjustable peripheral resistance, allowing regulation of the pulmonary pressure [15]. The outflow of the impedance simulator was connected to a reservoir which served as a preload for the right atrium. TV flow rate was measured with a transit time flow meter (HT110R, Transonic System, Inc., Ithaca, NY, USA), equipped with a 1″ probe placed upstream from the atrial connector. Pulmonary, ventricular, and atrial pressures were measured by piezoresistive transducers (143PC03D model, 140PC series, Honeywell, Inc., Morristown, NJ, USA). All signals were acquired with an A/D converter (DAQ USB 6210, National Instruments, Austin, TX, USA) at sampling frequency of 200 Hz.

The mock loop was set to obtain the following working conditions:

- Heart rate (HR): 60 bpm;
- Pump stroke volume (SV): 90 ml;
- Mean right atrial pressure (RAP): 10 mmHg;
- Mean pulmonary artery pressure (PAP): 20 mmHg (normotensive), 40 mmHg (mild hypertensive), and 60 mmHg (severe hypertensive,) according to the ISO 5910:2018.

### Haemodynamic Assessment

**Fig. 3:**
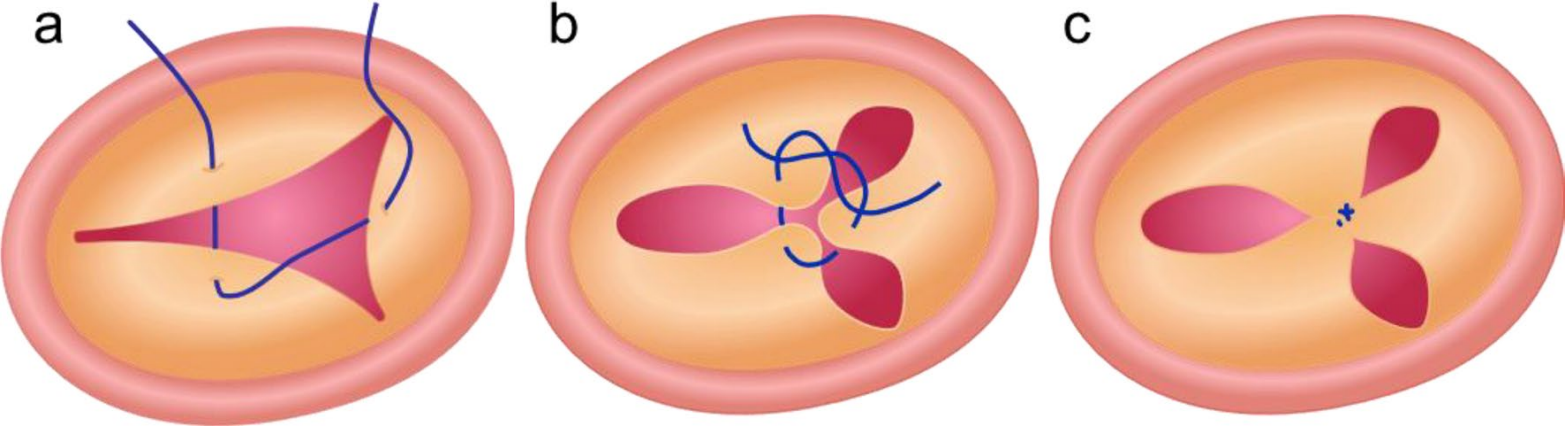
Surgical steps of the clover technique

Haemodynamic assessment was performed on 6 porcine heart samples (weight 516 ± 29 g). The following cases were analysed and compared: FTR (pathological model), Clover technique (state-of-the-art treatment), and STD treatment. The pathological model obtained in the ex vivo setup represented the baseline case; Clover technique was reproduced by an expert surgeon, suturing TV leaflets as shown in Fig. 3. The three cases were tested in randomized sequence in each sample.

For the haemodynamic assessment, working parameters were set to simulate normotensive and mild hypertensive condition, and the following haemodynamic indexes were obtained from the raw data:

- ΔP_diast_mean (mmHg)—mean pressure drop across TV during diastole;
- TV Flow (L/min)—mean tricuspid flow rate,
- TV_BF (ml)—tricuspid valve regurgitant volume - evaluated as time integral of the negative portion of the flow rate curve;
- TV_BF reduction (%)—percentage of backflow volume reduction evaluated as follows:

$$\frac{{{\text{TV}}_{\text{BF}}}_{\text{baseline}}-{{\text{TV}}_{\text{BF}}}_{\text{post treatment}}}{{{\text{TV}}_{\text{BF}}}_{{\text{baseline}}}}\times 100.$$

### Force Transducers

**Fig. 4:**
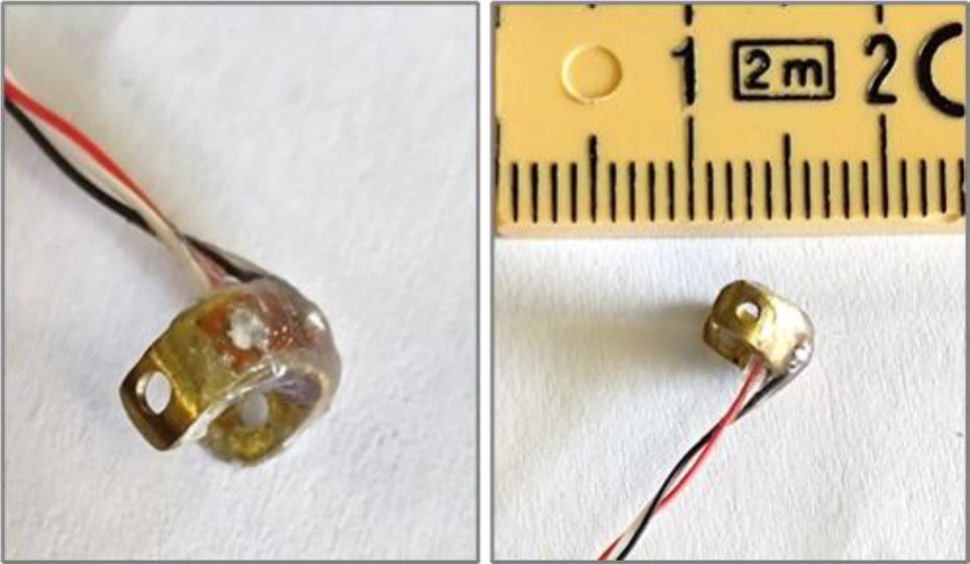
In-house-developed load cell

In-house-developed force transducers (Fig. 4) were adopted [16, 17] for the measurement of device–tissue interaction force. Two 350-Ω strain gauges in half-Wheatstone bridge configuration were mounted on a C-shaped brass frame (5 mm inner diameter, 6 mm outer diameter, 4 mm width). Transducers were calibrated and tested for linearity and stability over time in water before use.

### Forces Assessment

#### Extraction Force Tests

**Fig. 5:**
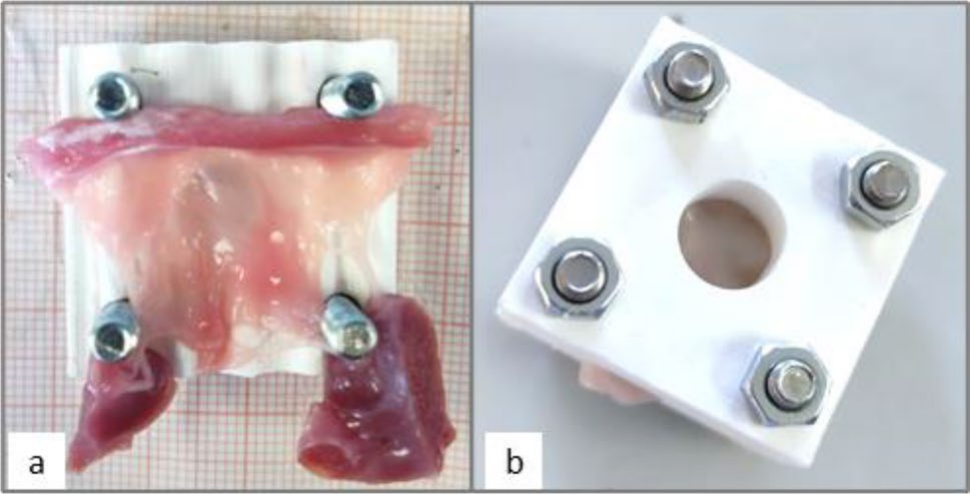
Tricuspid leaflet prepared for the test. **a** Leaflet on custom-made 3D-printed frame. **b** Closed frame with anchor channel

Tensile tests were conducted to characterize the force needed to completely extract the STD anchors from TV leaflets tissue. Tricuspid valves from 6 defrosted pig hearts (heart samples weighted 514 ± 76 g) were excised and the anterior, posterior, and septal leaflets were isolated and secured to a custom 3D-printed frame (Fig. 5) Suture wires with anchors were inserted into the leaflet and the frame was housed in a uniaxial tensile testing system (ELIS Z3-X1200) with a 200N load cell.

Destructive tensile tests with constant displacement speed of 60 mm/min were performed imposing a 0.1-N preload. Maximum forces corresponding to the extraction forces were recorded and compared to forces acquired in the pulsatile tests.

#### Interaction Force Tests

**Fig. 6:**
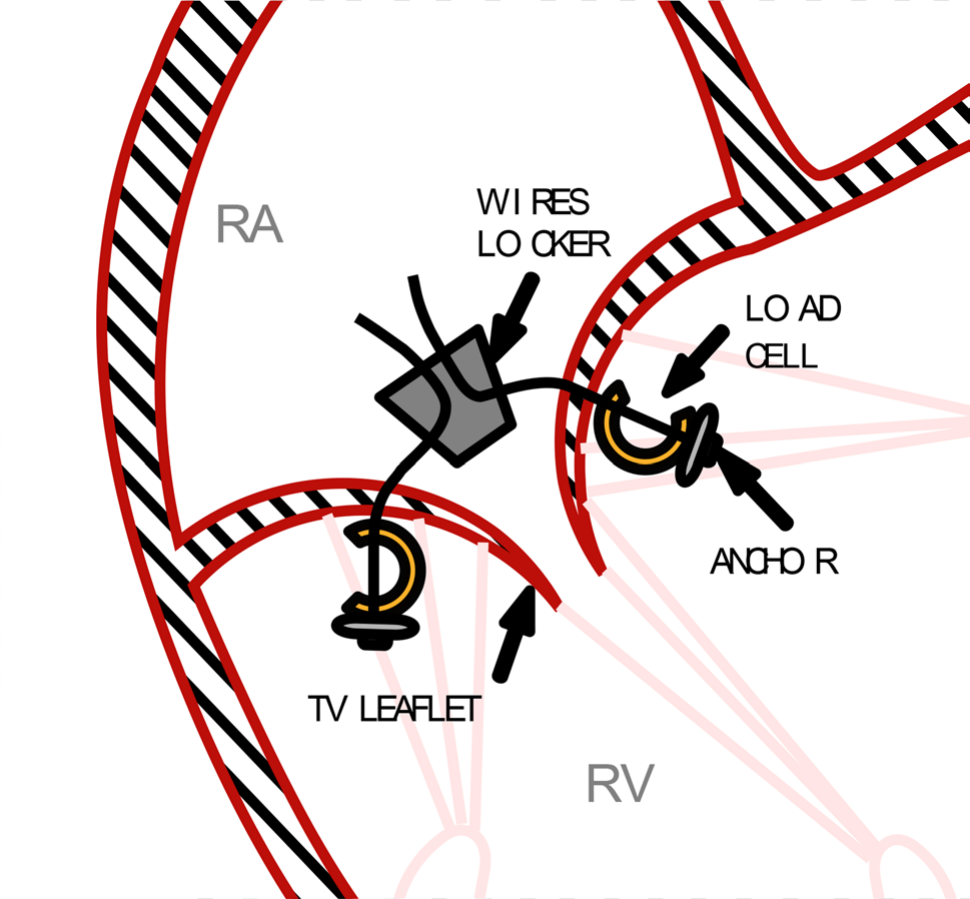
Schematic representation of the STD and load cell mounting

For assessment of STD–leaflet interaction forces, load cells and STD were implanted into TVs of 9 porcine heart samples (samples weighted 480 ± 28 g) as shown in Fig. 6. Suture insertion site was selected according to STD insertion protocol, and load cells were threaded into the suture wires and placed between the anchors and the leaflets.

Samples and load cells were connected to the pulsatile flow mock loop and acquisition system, respectively. Load cells were balanced to remove any zero drift and the device was secured imposing a slight compression to the load cells (0.1–0.8N). Force values due to the tightening of STD (*closing_offset*) were recorded and then pulsatile tests were performed. Normotensive, mild hypertensive, and severe hypertensive conditions were simulated for interaction force tests. Tests were repeated three times on each heart sample. Force signals were elaborated for each leaflet separately and the maximum force value was obtained.

### Statistical Analysis

Data were expressed as mean ± standard deviation. As regards the haemodynamic comparison, statistical differences between the different cases were assessed through a one-way Anova and Tukey’s multiple comparisons test. For force assessment test, statistical differences between loads recorded before and after treatments on each leaflet and in the different loading conditions were assessed using a two-way Anova and Tukey’s multiple comparisons test. *p* values < 0.05 were considered statistically significant.

## Results

### Haemodynamic Assessment

**Fig. 7:**
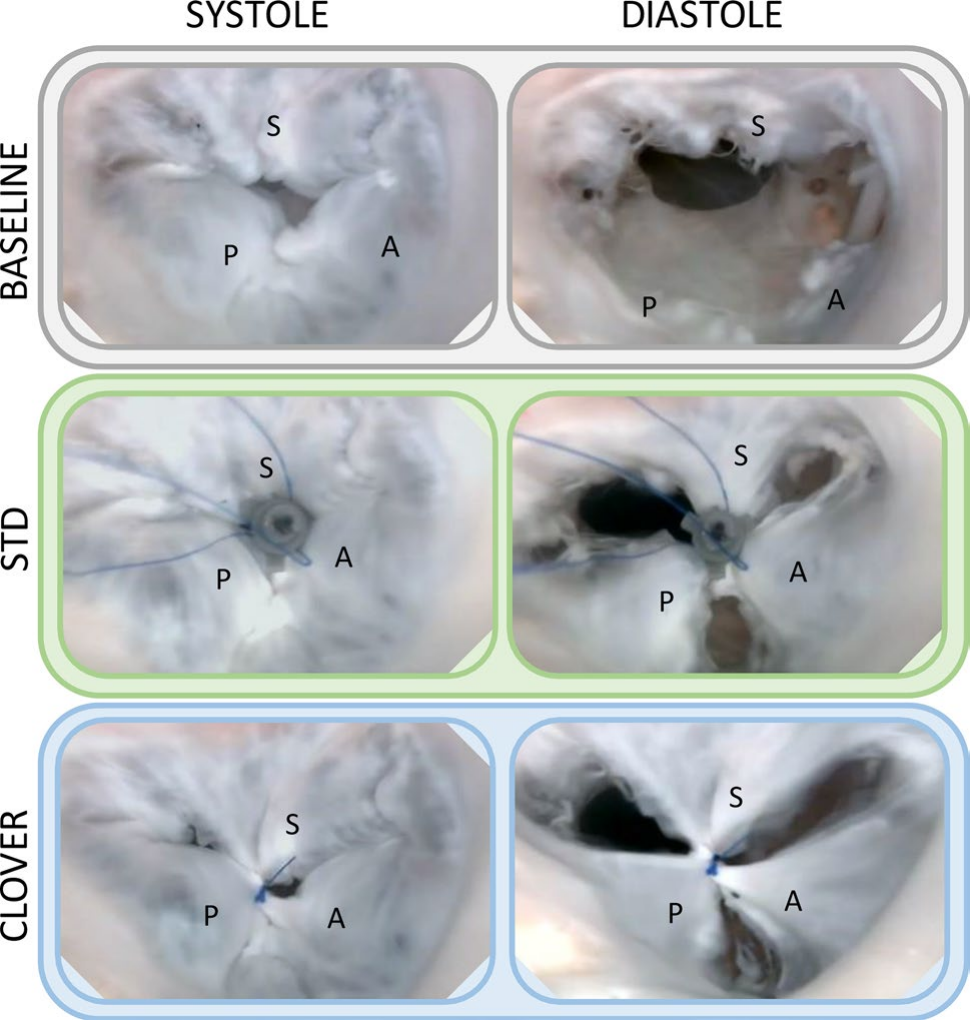
Endoscopic views of the TV in the tested cases in systolic and diastolic phase. S, A, P: septal, anterior, and posterior leaflet

Endoscopic views of a TV sample in the tested cases are reported in Fig. 7**.** Baseline central leakage and its reduction following treatment is visible in systole (left column), whilst clover configuration of the opening of the treated valve is shown in diastole (right column). Fluid dynamic indexes recorded in all tested conditions are reported in Table 1.

**Table 1:** Fluid dynamic indexes of the haemodynamic assessment

	Normotensive (PAP: 20 mmHg)						Mild hypertensive (PAP: 40 mmHg)					
	FTR	Clover	STD	*p* value			FTR	Clover	STD	*p* value		
				FTR vs Clover	FTR vs. STD	Clover vs. STD				FTR vs Clover	FTR vs. STD	Clover vs. STD
Mean PAP (mmHg)	20.5 ± 0.3	20.9 ± 1.3	20.4 ± 0.8	0.80	1.00	0.40	38.3 ± 4.1	40.1 ± 0.5	40.2 ± 0.5	0.92	0.85	0.94
Mean RAP (mmHg)	10.9 ± 1.1	11.6 ± 0.7	11.5 ± 0.8	0.06	0.08	0.87	11.0 ± 1.1	11.7 ± 0.7	11.5 ± 0.7	0.07	0.16	0.33
ΔP_diast (mmHg)	1.0 ± 0.5	2.1 ± 1.7	2.2 ± 1.6	0.25	0.17	0.80	1.3 ± 0.8	2.3 ± 1.6	2.4 ± 1.5	0.24	0.15	0.18
TV_Flow (l/min)	2.2 ± 0.8	3.2 ± 0.8	3.3 ± 0.6	0.01	< 0.001	0.79	1.0 ± 0.6	2.4 ± 0.6	2.4 ± 0.5	0.01	0.01	0.80
TV_ BF (ml)	62.8 ± 20.4	32.3 ± 11.4	33.2 ± 12.5	0.01	0.01	0.91	81.8 ± 28.0	42.1 ± 17.1	43.0 ± 17.6	0.01	0.01	0.97

Data are reported as mean value ± standard deviation (*n* = 6)

*Mean PAP* mean pulmonary artery pressure, *Mean RAP* mean right atrial pressure, *ΔP_diast* tricuspid transvalvular diastolic pressure, *TV_Flow* tricuspid flow rate, *TV_ BF* tricuspid backflow volume

**Fig. 8:**
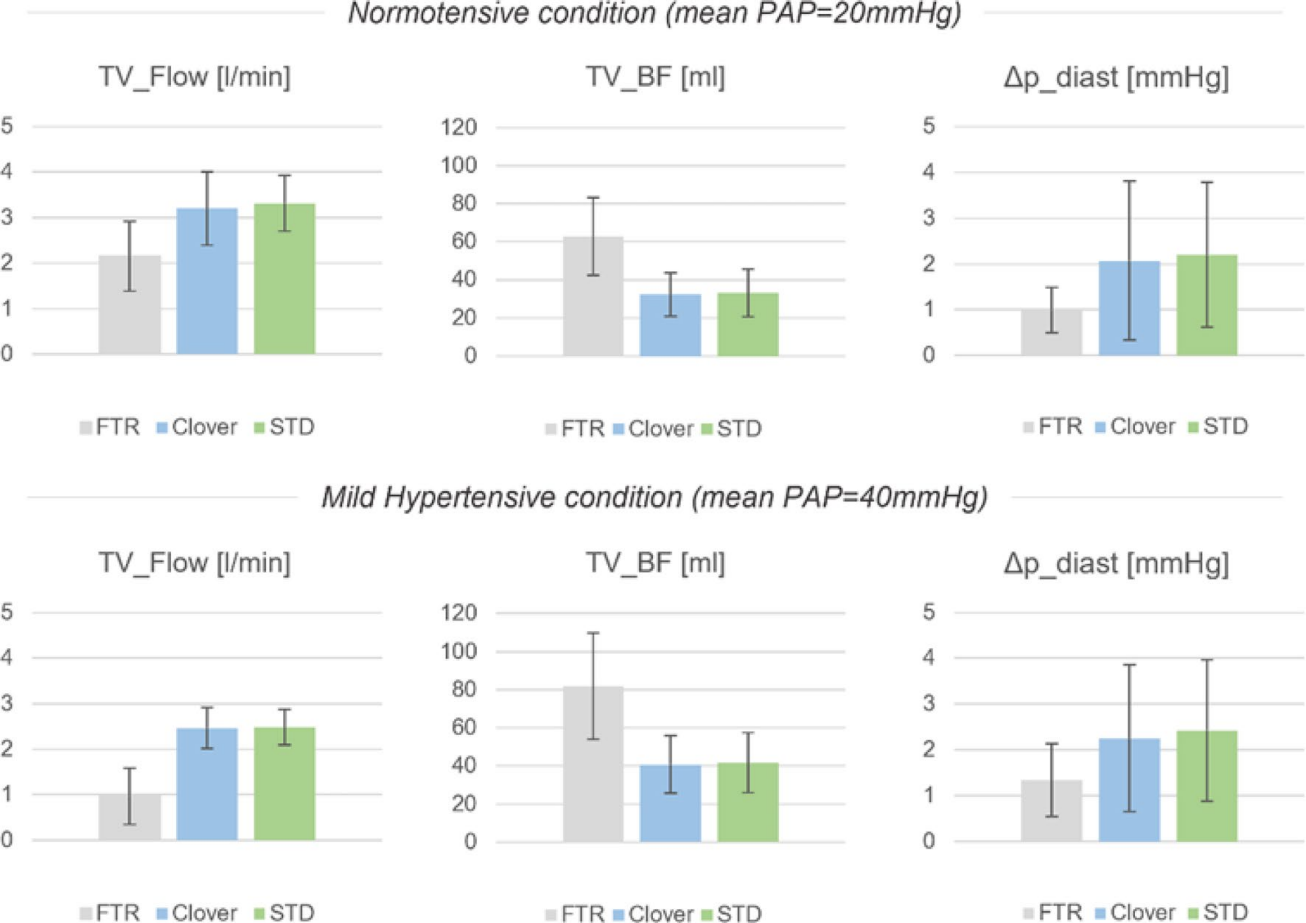
Comparison of indexes evaluated in the haemodynamic assessment

Figure 8 shows a comparison of haemodynamic indexes for the different working conditions in the three different cases. In the normotensive condition, TV_Flow significantly increased after the application of Clover technique and of STD, resulting in an average TV_BF reduction of 48% and 47%, respectively. The same trend was observed in the mild hypertensive condition.

In the normotensive condition, ΔP_diast_mean increased from 1.0 ± 0.5 mmHg (untreated FTR) to 2.1 ± 1.7 mmHg (*p* = 0.25) in the clover case and to 2.2 ± 1.6 mmHg (*p* = 0.17) in the STD case

In the mild hypertensive condition, the pathological value of 1.3 ± 0.8 reached a mean value of 2.3 ± 1.6-mmHg post-Clover application and of 2.4 ± 1.5 mmHg post-STD implantation highlighting the formation of a slightly and not statistically significant degree of stenosis.

### Forces Assessment

#### Extraction Force

Table 2 shows mean thickness and force values measured for the three leaflets.

**Table 2:** Force and thickness values recorded during extraction tests.

	Extraction force (N)	Thickness (mm)	*R* square
Septal	18.7 ± 6.7	0.37 ± 0.09	0.11
Anterior	22.5 ± 7.5	0.20 ± 0.03	0.21
Posterior	25.6 ± 9.4	0.31 ± 0.09	0.05

Data are reported as mean value ± standard deviation (*n* = 6)

Overall extraction force was 22.16 ± 8.6N. No statistically significant difference was found between the force recorded on septal, anterior, posterior leaflets, and no correlation was found between measured leaflet thickness and force values (*R*^2^ = 0.01909).

#### Interaction Force

**Fig. 9:**
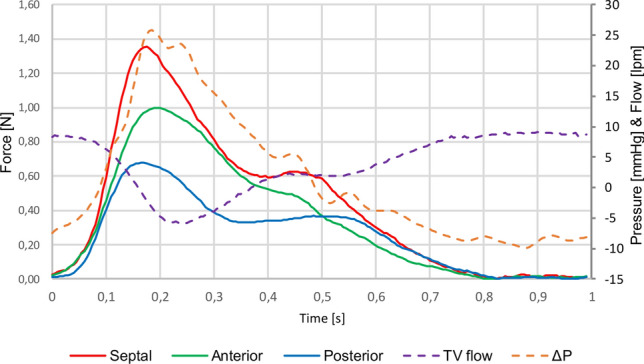
Representation of raw data acquired in time. Anterior, posterior, and septal: force value recorded by the load cell on the anterior, posterior, and septal leaflet; *ΔP* tricuspid transvalvular pressure, *TV flow* tricuspid flow rate

Fig. 9 shows a representative plot of the forces, TV flow, and PAP acquired in a cardiac cycle. The forces recorded were compressive all over the cardiac cycle. The force plot showed a peak in the systolic phase, in correspondence of the maximum value of transvalvular pressure and of the negative peak of the tricuspid backflow. Force values recorded in normotensive, mild hypertensive, and severe hypertensive conditions are reported in Table 3.

**Table 3:** Values of the compression force recorded in normotensive, mild, and severe hypertensive conditions

Max force (N)				*p* value (Anova)	
	Normotensive (PAP = 20 mmHg)	Mild hypertensive (PAP = 40 mmHg)	Severe hypertensive (PAP = 60 mmHg)	Pressure	Leaflet
Septal leaflet	1.3 ± 0.41	1.5 ± 0.48	1.7 ± 0.52	< 0.001	0.1921
Anterior leaflet	1.0 ± 0.32	1.2 ± 0.38	1.4 ± 0.47		
Posterior leaflet	1.1 ± 0.26	1.2 ± 0.37	1.3 ± 0.45		

Values are expressed as mean ± standard deviation (*n *= 9)

**Fig. 10:**
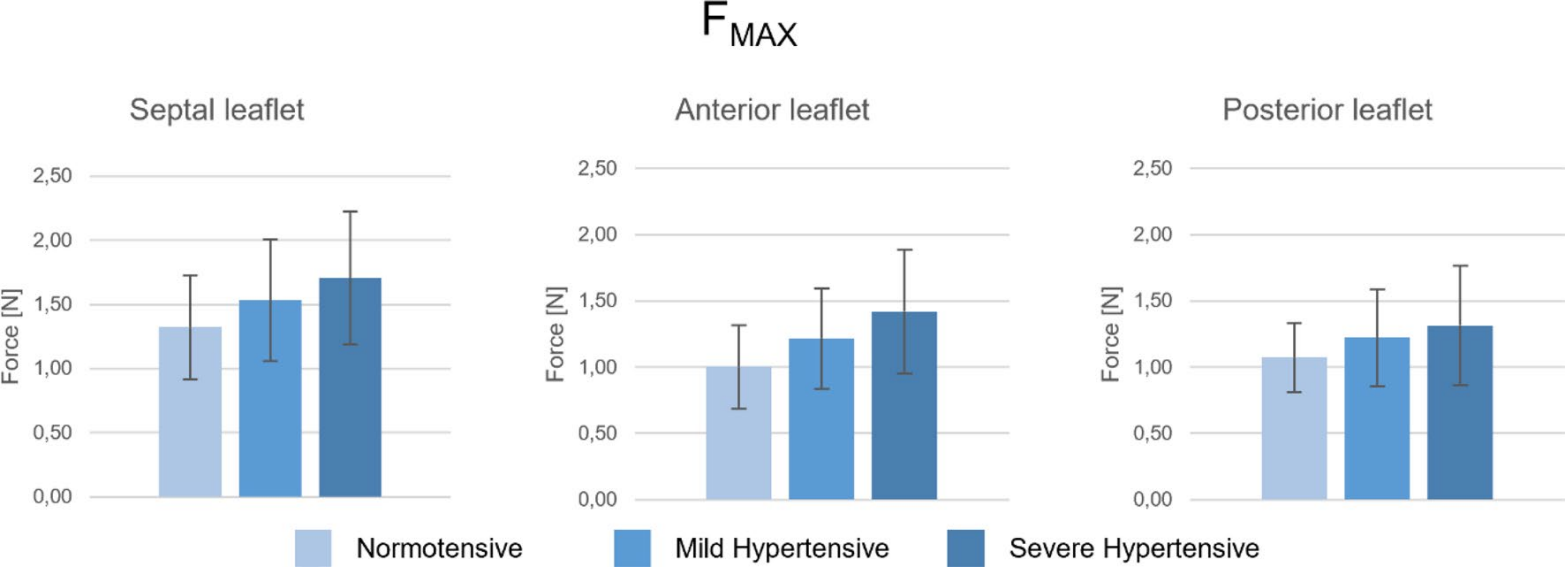
*F*_max_ recorded on each of the TV leaflet in the imposed working conditions (Normotensive, Mild Hypertensive, and Severe Hypertensive condition). Values are represented as mean value; error bars show standard deviation

Figure 10 shows a comparison of *F*_max_ recorded in the different working conditions for each TV leaflet. *F*_max_ values ranged from 1N to 1.71N. Highest values of Fmax were recorded on the septal leaflets. *F*_max_ values increased linearly with mean PAP (*R*^2^ = 0.97–0.99). Statistically significant differences (*p* value < 0.0001) were found amongst the different working conditions (mean PAP: 20-40-60 mmHg) for each leaflet. No statistically significant difference was found amongst the force values on different leaflets in the same working condition (*p* value > 0.05). Furthermore, Fmax evaluated for each leaflet and conditions was systematically lower than the extraction force.

## Discussion

In this work, we presented a preliminary evaluation of a novel approach for the treatment of TR. The approach is based on a single device able to grasp and approach the TV leaflets towards the centre of the valve to fill the coaptation gap, thus replicating the well-established Clover surgical approach [4]. We assessed the haemodynamic performance of the STD in an experimental ex vivo model of FTR where we compared fluid dynamic indexes of a TV pathological model to the ones following STD and clover surgical technique application. Moreover, we presented a preliminary safety investigation aimed at evaluating the forces acting between STD and the biological structures.

The ex vivo approach here adopted replicated the pathological valve, treated and evaluated, in terms of haemodynamic and forces, in the immediate post-procedural scenario. In this setting, STD showed similar effectiveness to the surgical approach in counteracting TR, restoring TV haemodynamic. The exploratory force characterization suggested a safe interaction of STD with the biological structures.

In the haemodynamic assessment, we compared the effects of STD and of Clover technique on an experimental model of FTR, investigating the alterations in TV flowrate and in diastolic transvalvular pressure following treatments. The application of the two approaches led to a significant (*p* = 0.01) reduction of TV backflow, when compared to the pathological model. Moreover, the two strategies showed equivalent efficacy in backflow reduction in both normotensive (*p* = 0.70) and mild hypertensive (*p* = 0.97) experimental conditions. As expected, we observed an increase in diastolic transvalvular pressure following the treatments, since both STD and Clover are inherently obstructive and induce a reduction of the valvular orifice. Of note, this alteration was similar in the two treatments (*p* = 0.8 and *p* = 0.2 in normotensive and in mild hypertensive condition, respectively) and was not significant with respect to the baseline condition. When comparing with clinical data, mean diastolic transvalvular pressure values were close to the one experienced with the clover technique [18] and were lower than the clinical indication for tricuspid stenosis [19], this suggesting no risk of serious valve occlusion. The use of miniaturized force transducers enabled for the characterization of the tensional state of the STD in different working conditions. The force signal varied in time following the trend of transvalvular pressure and TV flow rate, peaking in the simulated systolic phase, in correspondence of the maximum of pressure and TV backflow. The values of maximum force showed a significant variation with the simulated working condition (normotensive, mild, and severe hypertensive), highlighting a major dependence of these forces on the ventricular pressure. These forces were compared to those recorded in specifically designed mechanical tests resulting in a magnitude range ten times lower than the one necessary for STD anchors to completely pass through the leaflets. This suggests that no relevant and immediate damage of the leaflet would occur following STD implant. We identified the extraction force of the anchor as the most relevant failure mode to compare to the interaction forces. Indeed, if correctly implanted, the device should acquire a configuration with the anchors being completely in contact with the wire locker and leaflets trapped within the two components. In this case, the main failure mode would be the anchor perforating the leaflet. However, it is not possible to exclude the risk of the suture wire tearing the leaflet, so further investigations have to be performed to get an overall comprehension of all the possible failure phenomena.

Moreover, the force values recorded during interaction tests were affected by the presence of the force transducers which increased the overall mass of the device and so its inertial effects. This reasonably led to an overestimation of the forces increasing the safety of the investigation.

It has to be noted that in this study, both STD and surgery were applied in absence of annuloplasty technique, which is usually applied together with the Clover to treat TR [18]. Nonetheless, several edge-to-edge procedures aimed at FTR treatment are reported in literature to be applied without annuloplasty [12, 20]. So, being the device intended to be a stand-alone treatment strategy, the experimental evaluation was designed to directly compare the effects of STD and of Clover technique without annuloplasty. It is also worth mentioning that these preliminary tests were conducted on an ex vivo platform which is limited to the representation of acute pathological and immediate post-treatment scenarios. Therefore, additional testing is mandatory to monitor the interaction and effects of device over time to ensure the effectiveness and reliability of the technology in the mid-long term. Nonetheless, it is not unlikely that the improvement in TV haemodynamic resulting from STD implant would induce a reduction of RV volume overload with a possible reverse remodelling of the ventricle in time [9, 21]. This might lead to a decrease in the tension between leaflets and STD improving device safety. Furthermore, the haemodynamic assessment was only performed in normotensive and mild hypertensive conditions due to inability to reproduce the severe hypertensive condition in the baseline case. The baseline case (untreated sample with TR) showed a major regurgitation which did not allow to reach a pulmonary artery pressure (PAP) representative of the severe hypertensive condition.

Finally, the scope of the investigation was to verify the feasibility, the efficacy, and the safety of the design concept represented by the prototypes of the implantable components of the STD technology. However, from the very beginning of development, together with the development of the implantable portion of the STD system, feasibility studies of the delivery device were conducted, taking into account aspects related to the safety of the delivery and the procedure as well as its usability. These studies demonstrated the technological feasibility of the delivery and in the future, during the next steps of development, will limit the redesign of the implantable components of the STD system and of the procedure itself. Despite its exploratory nature, this study highlights the potential of the STD technology in the treatment of FTR. This novel approach could potentially improve patients’ outcomes and, in perspective, reduce the need for invasive surgical interventions.
